# How is tree growth rate linked to root functional traits in phylogenetically related poplar hybrids?

**DOI:** 10.1093/treephys/tpae120

**Published:** 2024-09-16

**Authors:** Toky Jeriniaina Rabearison, Vincent Poirier, Jérôme Laganière, Annie DesRochers

**Affiliations:** Institut de Recherche sur les Forêts, Université du Québec en Abitibi-Témiscamingue, 341 rue principale Nord, Amos, Québec J9T 2L8, Canada; Unité de recherche et de développement en agroalimentaire, Université du Québec en Abitibi-Témiscamingue, 79, Rue Côté, Notre-Dame-du-Nord, Québec J0Z 3B0, Canada; Natural Resources Canada, Canadian Forest Service, Laurentian Forestry Centre, 1055 du P.E.P.S., PO Box 10380, Stn Sainte-Foy, Québec, Québec G1V 4C7, Canada; Institut de Recherche sur les Forêts, Université du Québec en Abitibi-Témiscamingue, 341 rue principale Nord, Amos, Québec J9T 2L8, Canada

**Keywords:** fast-growing tree, fine root, hybrid poplar, root economics spectrum, subsoil, tree productivity

## Abstract

Fine roots play a crucial role in soil nutrient and water acquisition, significantly contributing to tree growth. Fine roots with a high specific root length (SRL) and small diameter are often considered to help trees grow fast. However, inconsistencies in the literature do not provide a clear basis on the effect of root functional traits, such as SRL or root mass density (RMD), on tree growth rate in phylogenetically related trees. Our aim was to examine relationships between tree growth rate and root functional traits, using clones displaying different growth rates in a hybrid poplar plantation located in New Liskeard, ON, Canada. Fine roots (diameter < 2 mm) samples were collected using soil cores at depths of 0–20, 20–40 and 40–60 cm, and analyzed for morphological, chemical and architectural traits. High SRL and thin fine roots were associated with the least productive clones, which is not consistent with the root economics spectrum (RES) theory. However, the most productive clone had larger fine root diameter and higher root lignin concentrations, probably reducing root construction and maintenance costs and carbon losses. Therefore, at the 0–20 and 20–40 cm depths, tree growth rates showed positive correlations with root diameter and root lignin concentrations, but negative correlations with SRL and root soluble compounds concentration. Increasing RMD at the 0–20 cm depth promoted tree growth rates, showing the importance of soil exploration in the topsoil for tree growth. We conclude that fine root variation does not always follow the RES hypothesis and argue that the rapid growth rate of trees may also be driven by fine root growth in diameter and mass in phylogenetically related trees.

## Introduction

Fast-growing plantations could help meet the world’s growing demand for harvested wood products by rapidly producing high woody biomass on small land areas. For instance, hybrid poplars (*Populus* spp.) can grow three times higher than spruce trees of similar age in the boreal and temperate climate of Canada (Peichl et al. 2006; Chomel et al. 2014). Even among hybrid poplar clones, a great variability in growth rates can be observed as they can originate from different parentage (Laureysens et al. 2004; Truax et al. 2014). One of the key factors behind these differences is fine root development, because they play a central role in soil resource acquisition (McCormack et al. 2015). Fine roots are usually defined as the most distal, ephemeral and absorptive roots less than 2 mm in diameter (D) (Guo et al. 2008, Ostonen et al. 2017). To better understand the influence of fine roots (D < 2 mm) on tree growth rates, a functional trait-based approach is often applied in the literature (Cadotte et al. 2009; Roscher et al. 2012; Bardgett et al. 2014). Understanding how growth strategies are linked to functional root traits could help improve management and selection of clones/varieties to establish fast-growing plantations.

According to the root economics spectrum (RES) hypothesis, morphological and chemical traits of fine roots show interspecific variation depending on the plant’s growth strategy (McCormack et al. 2012; Bardgett et al. 2014; Reich 2014; Roumet et al. 2016). Although the ‘acquisitive’ strategy is said to enhance rapid tree growth with faster foraging, thinner and shorter lifespan roots, the ‘conservative’ strategy is related to slower foraging, and thicker and longer lifespan roots (Comas et al. 2002; McCormack et al. 2012; Reich 2014; Kramer-Walter et al. 2016). Previous studies report that fast-growing trees exhibit higher specific root length (SRL), root nitrogen (N) concentrations, and smaller root diameters than slow-growing species (Wright and Westoby 1999; Comas et al. 2002; Comas and Eissenstat 2004; Roumet et al. 2016). Fine roots with high SRL would be more efficient for tree growth as more carbon (C) is allocated into fine root elongation than in biomass, increasing absorption capacity (Eissenstat and Yanai 1997). However, high SRL and thin fine roots are often associated with short root lifespan and high root respiration rates, which increases C costs of fine root maintenance and C losses through respiration, negatively impacting overall net C gain of trees (Pregitzer et al. 1998; Makita et al. 2009; McCormack et al. 2012; Weemstra et al. 2020). Consequently, the positive relationship between increased SRL and tree growth remains unclear.

Several studies showed that SRL, root diameter and root N concentrations of tree species did not always correlate with tree growth rates (Kramer-Walter et al. 2016; Weemstra et al. 2016, 2020) as nutrient acquisition in soils does not necessarily require root traits a priori defined as acquisitive but also other root traits linked to soil nutrient mobility (Ravenek et al. 2016). Among architectural traits of fine roots, RMD or root length density (RLD), i.e. fine-root mass or length per unit soil volume, determines the soil exploration intensity of roots (Stokes et al. 2009; Pérez-Harguindeguy et al. 2013). Increasing RMD leads to an increase in root surface area available to efficiently absorb mobile and less mobile nutrients, and thus to improve tree growth rates (Bauhus and Messier 1999; Finér et al. 2011; Hansson et al. 2013). However, there is still a lack of studies confirming the effects of these root traits on the rapid growth of some trees such as hybrid poplars. According to Weemstra et al. (2020), tree species can use two distinct strategies to increase root surface area and soil nutrient uptake: allocate more C into root growth to increase RMD of fine roots or develop longer fine roots (higher SRL). It remains unclear whether acquisition and architectural traits of fine roots vary in a coordinated way or play a separate role in reaching faster growth rates in plantations.

Variation in fine root traits may not necessarily follow the RES hypothesis but be explained solely by the fact that studied tree species belonged to different orders or groups, for example conifers versus broadleaves (Tobner et al. 2013; Valverde-Barrantes et al. 2015; Lwila et al. 2021). In temperate tree species, Valverde-Barrantes et al. (2015) argued that the substantial differences in root traits between species groups can cause profound divergences in resource acquisition strategies among them. In order to minimize the potential effect of high-level phylogenetic differences on the relationship between aboveground growth rates and fine-root traits, our study focused exclusively on species belonging to the same genus but with different growth rates. We studied hybrid poplar clones from different parentages considered as different species but belonging to the same genus *Populus*, thereby minimizing phylogenetic differences.

In this research, we aimed to compare fine root traits between hybrid poplars clones having different growth rates, to examine the relationships between aboveground growth rate and morphological, chemical and architectural traits of fine roots in phylogenetically related trees. We used the arbitrary but commonly applied definition of fine roots as those less than 2 mm in diameter. As previous studies found that tree fine roots (D < 2 mm) can include several branching orders that differ in morphology and function (Pregitzer et al. 1998; Pregitzer et al. 2002; McCormack et al. 2015), we further divided our fine roots into three diameter classes: < 0.2, 0.2–1 and 1–2 mm. In line with the RES theory, we hypothesized that SRL, root N and soluble compounds concentrations would be positively correlated to tree growth rates, whereas root diameter would have the opposite effect. Great root length and mass densities give trees a high intensity of soil exploration to acquire nutrients; hence, we predicted that they would increase tree growth rates.

## Materials and methods

### Study site

The study was conducted in an experimental plantation of hybrid poplars at the New Liskeard Agricultural Research Station in North-Eastern Ontario, Canada (47°31′15″ N, 79°39′52″ W). The area is characterized by a humid continental climate with an average daily temperature of 2.6 °C and an average annual precipitation of 786 mm (576 mm rain and 222 cm snow) based on 29-year data (1981–2010, Earlton station) (Environment Canada 2023). The soil in this region has a clay loam texture and is classified as a Humic Gleysol (Canada Soil Survey Committee 1987, Yan et al. 2019, Rabearison et al. 2023).

The site was ploughed and cross-cultivated with agricultural disks in October 2006, followed by pre-emergent herbicide applications in spring 2007. One-year-old hybrid poplar rooted cuttings were planted in spring 2007 with the spacing of a 3.5 m × 3.5 m (816 stems ha^−1^). All trees were spot fertilized solely at planting with NPK 18-23-18 (110 g tree^−1^), at a rate of 89.76 kg ha^−1^ for optimal initial growth, without additional fertilization over the years. The experimental design consisted of three replicate blocks, each containing eight monoclonal plots of 100 trees (10 rows × 10 trees) randomly distributed within blocks. We controlled the presence of weeds by cultivating between rows with disks followed by herbicide applications between trees (RoundupTM) for the first 2 years after plantation establishment.

### Clone selection and growth rate

After 14 growing seasons, we selected five clones from the eight planted ones based on their growth rates and their parentage, ranging from the least to the most productive: 747210 (*P. balsamifera* × *P. trichocarpa*), 915005 (*P. balsamifera* × *P. maximowiczii*), 1079 *(Populus* × *jackii* (*P. balsamifera* × *P. deltoides*)), 915319 (*P. maximowiczii* × *P. balsamifera*) and DN2 (*P. deltoides* × *P. nigra*). For each monoclonal plot, we measured diameter at breast height of trees to estimate the growth rate of each clone. Average tree characteristics (diameter at breast height, tree height and stem volume) for each clone can be found in Rabearison et al. (2023). Tree growth rate (Table 1) was calculated as total stem volume divided by plot area and plantation age (Truax et al. 2012; Truax et al. 2014).

**Table 1:** Growth rates and labels of studied hybrid poplar clones (Rabearison et al. 2023).

Clone	Mean growth rate (m^3^ ha^−1^ year^−1^)		Label
747210	12.58 (±0.92)	d	Least productive
915005	16.04 (±0.61)	c	Second least productive
1079	19.49 (±0.57)	b	Mid-productive
915319	21.23 (±0.78)	b	Second most productive
DN2	24.25 (±0.20)	a	Most productive

Standard errors of the mean (±SEM) are given in parentheses. Different letters indicate significant differences between clones (*P* < 0.05).

### Root sampling

Sampling was systematically done between two trees in each monoclonal plot in July 2021. Soil cores were collected at two distances (87.5 and 175.0 cm) from a stem and at three soil depths (0–20, 20–40 and 40–60 cm) using a polyvinyl chloride cylinder measuring 10 cm in diameter and 20 cm in length. We had six replicates (two trees per block × three blocks) for each clone, distance and depth. After being stored in plastic bags and a cooler, a total of 180 samples (six replicates × two distances × three soil depths × five clones) were brought to the laboratory for further analysis. We soaked the soil cores in water containing sodium hexametaphosphate (50 g L^−1^) overnight to disperse soil particles adhering to the roots due to the clay loam texture of the soil (Smucker et al. 1982; Yan et al. 2019). Roots were washed and rinsed to completely remove soil particles using a hydropneumatic elutriation system (Root washer, Gillison's Variety Fabrication Inc., Benzonia, MI, USA) according to Smucker et al. (1982). The roots were visually sorted to select only living and fine roots (D < 2 mm) of hybrid poplars for further analysis. While living fine roots were more rigid and flexible, dead roots were fragile, darker in color and broke easily. We were unable to collect enough roots from a soil core at the 40–60 cm depth to carry out all root analyses, so we had to mix root samples from two trees of the same clone, the same distance and the same block at this depth. A total of 150 root samples were available for analysis, including 60, 60 and 30 samples from depths of 0–20, 20–40 and 40–60 cm, respectively.

### Root trait analysis

All washed fine roots were spread into a 20 × 25 cm transparent tray filled with deionized water to minimize overlap between roots and scanned as grayscale images at 400 d.p.i. resolution with a transmitted light source (Epson Perfection V800; Epson, Ontario, Canada). We subsequently used WinRhizo Pro 2019 software (Regent Instruments, Quebec, Canada) to determine average root diameter (D, mm), total root length (RL), root length of three diameter classes (< 0.2, 0.2–1 and 1–2 mm) and root volume. We calculated the percentage in length of each root size class by multiplying the length of each class by 100 and dividing by RL. It should be noted that we were unable to determine the mass for each root size class as it was not possible to separate and weigh the different root size classes manually. Thus, we measured SRL values for whole fine roots (D < 2 mm), but not for each root size class. All scanned and weighed roots were then oven dried for 48 h at 60 °C to determine dry mass of whole fine roots (RDM). SRL (m g^−1^) was calculated as the ratio of total root length to RDM. Root dry matter content (RDMC, mg g^−1^) was obtained as RDM divided by root fresh mass (RFM). Root tissue density (RTD, g cm^−3^) was calculated as RDM divided by root volume. We calculated RMD (g cm^−3^) as the ratio of the total root dry mass (RDM) to the soil volume where the roots were extracted. RLD (cm cm^−3^) was obtained by multiplying RMD by SRL.

Dried root samples were finely ground using a 2 mm sieve and an ultra-centrifugal mill (ZM200, Retsch GmbH, Haan, Germany). We analyzed root carbon (RCC) and nitrogen (RNC) concentrations by dry combustion (Vario MAX cube; Elementar, Langenselbold, Germany). Water-soluble compounds, hemicelluloses and lignin concentrations (mg g^−1^) of fine roots were obtained by the method of Van Soest et al. (1991) using a fiber analyzer (Fibersac 24; Ankom, Macedon, NJ, USA).

### Statistical analysis

**Figure 1:**
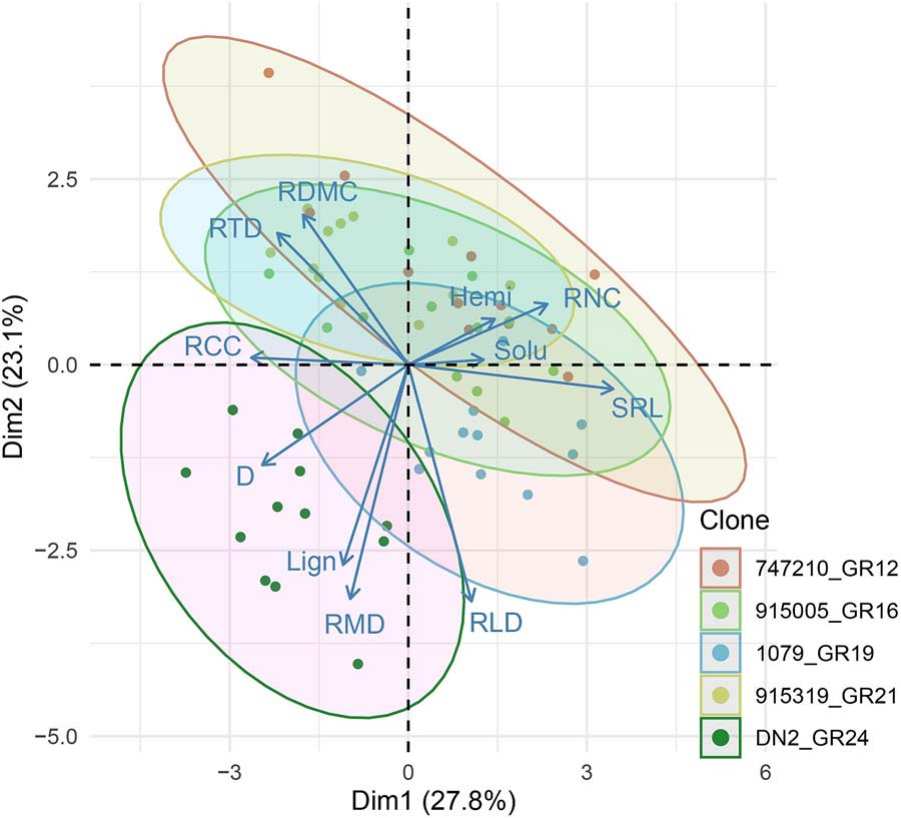
PCA of fine-root traits at 0–20 cm depth. Each colored region represents a labeled clone with its parentage coding and growth rate (GR, m^3^ ha^−1^ year^−1^) and has a confidence interval of 95%. D: average root diameter, hemi: root [hemicellulose], Lign: root [lignin], RCC: root [carbon], RDMC: root dry matter content, RLD: root length density, RMD: root mass density, RNC: root [nitrogen], RTD: root tissue density, Solu: root [soluble compounds] and SRL: specific root length.

All statistical tests were done using R software version 4.2.2 (R Development Core Team 2013). We first used principal component analyses (PCA) to obtain an overview of the multidimensional variation of fine-root traits and to select all root traits with best significant contributions by using *prcomp* function and factoextra package. As variations in root traits were notably less pronounced at the 20–40 and 40–60 cm depths than at the 0–20 cm depth (Fig. 1 and Fig. S1 available as Supplementary data at *Tree Physiology* Online), statistical analyses were carried out separately at each depth. Selected root traits were compared between clones (six replicates) at each depth by using linear mixed models where block was considered as random effect (lme4 and lmerTest packages, Bates et al. 2014; Kuznetsova et al. 2017). The predictor effect was significant when the probability level (*P*) was below the theoretical probability level α = 5%. Model assumptions (independence of residuals, equality of variance (homoscedasticity) and normality of residuals) were tested by diagnostic graphs and Shapiro–Wilk tests and the assumptions of selected models were met without data transformation. The emmeans package in R was used as a post-hoc method to make pairwise comparisons when there were significant differences in root traits between clones (Lenth et al. 2018).

Relationships between tree growth rates and root traits were determined using linear mixed models. We selected models with smaller Akaike Information Criterion (AICc) by *aictab* function and AICcmodavg package (Table S1, Table S2 and Table S3 available as Supplementary data at *Tree Physiology Online*) and only the best root trait models were presented. Logarithmic transformations were performed for variable RMD at the 0–20 cm depth to meet model assumptions. We also performed linear mixed models with two root traits as predictors to define the best model, using *lmer* and *aictab* functions, while avoiding collinear predictors (i.e. RMD and RLD). Two coefficients of determination can be used in linear mixed models: the marginal R^2^ and the conditional R^2^ which are, respectively, the variance explained by the fixed effect (trait) and the variance explained by the fixed effect (trait) and random effect (block); we used only the marginal R^2^ (shown as R^2^ in this study) as it only considers the root trait effect (*r.squaredGLMM* function, MuMin package, Barton 2009; Nakagawa and Schielzeth 2013). The effects of distance from trees were not significant for all root traits hence were dropped, but data from both distances were retained to determine clone effects and the relationship between tree growth rate and root traits.

## Results

### Variations in root traits between hybrid poplar clones

Root traits among hybrid poplar clones showed multidimensional variations at the 0–20 cm depth according to PCA (Fig. 1). The first PCA axis accounted for 27.8% of overall variation and was related to SRL, D, RCC and RNC, while the second PCA axis explained 23.1% of overall variation and mainly reflected differences in RMD, RLD, root lignin concentration and RDMC. Net differences in fine root traits between the most productive clone (DN2) and the least productive one (747210) were observed along these two PCA axis (Figure 1).

**Figure 2:**
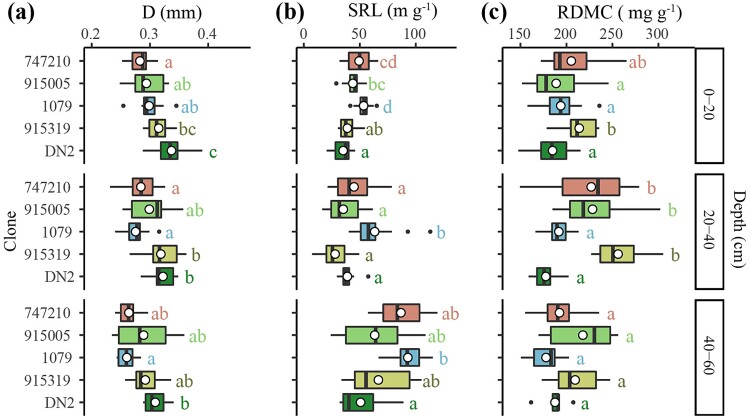
Differences in (a) average root diameter (D), (b) specific root length (SRL) and (c) root dry mass content (RMDC) between clones at each depth. Boxplots include the median (black vertical lines) and the mean (white circles) of each root trait for each clone at each depth. Different letters indicate significant differences between clones at each depth (*P* < 0.05). Clones are arranged in ascending order of their growth rates, from top to bottom.

The most productive clones (DN2 and 915319) produced thicker fine roots than the least productive clone (747210) at the 0–20 and 20–40 cm depths (*P* < 0.001 for both soil depths, Figure 2a). At the 40–60 cm depth, average fine root diameter of the most productive clone (DN2) exceeded that of the mid-productive clone (1079) (*P* < 0.05, Figure 2a). In contrast, the most productive clones (DN2 and 915319) had lower SRL than the mid-productive clone (1079) and the least productive clone (747210) at the 0–20 depth (*P* < 0.001, Figure 2b). The mid-productive clone (1079) had the highest SRL at the 20–40 cm depth (*P* < 0.001) and had higher SRL compared with clone DN2 at the 40–60 cm depth (*P* < 0.05) (Figure 2b). Furthermore, the second most productive clone (915319) had higher RDMC, i.e., dry mass divided by fresh mass of fine roots, than the most productive clone (DN2) and clone 1079 at the 0–20 and 20–40 cm depths (*P* < 0.001 for both soil depths, Figure 2c). RDMC values were not significantly different between clones at the 40–60 cm depth (Figure 2c).

**Figure 3:**
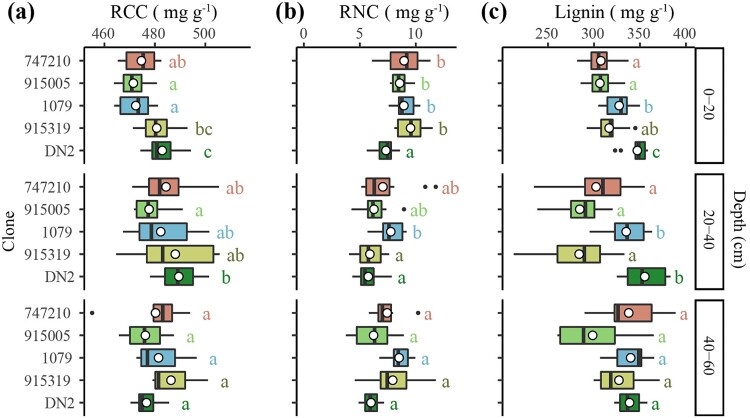
Differences in (a) root carbon concentration (RCC), (b) root nitrogen concentration (RNC) and (c) root lignin concentration between clones at each depth. Boxplots include the median (black vertical lines) and the mean (white circles) of each root trait for each clone at each depth. Different letters indicate significant differences between clones at each depth (*P* < 0.05). Clones are arranged in ascending order of their growth rates, from top to bottom.

In terms of chemical trait differences, fine roots of the most productive clones (DN2 and 915319) had higher C concentrations than those of the mid-productive clones (1079 and 915005) at the 0–20 cm depth (*P* < 0.001; Figure 3a). Root C concentrations were also greater for the most productive clone DN2 than for clone 915005 at the 20–40 cm depth (*P* < 0.05, Figure 3a). However, the most productive clone (DN2) had the lowest root N concentrations at the 0–20 cm depth (*P* < 0.001) and lower root N concentrations than the mid-productive clone (1079) at the 20–40 cm depth (*P* < 0.01) (Figure 3b). The highest lignin concentrations were observed in fine roots of the most productive clone (DN2) at the 0–20 cm depth, as well as in those of this clone and clone 1079 at the 20–40 cm depth (*P* < 0.001 for both soil depths, Figure 3c). At the 40–60 cm depth, there were no significant differences between clones for root C, N and lignin concentrations (Figure 3a–c).

**Figure 4:**
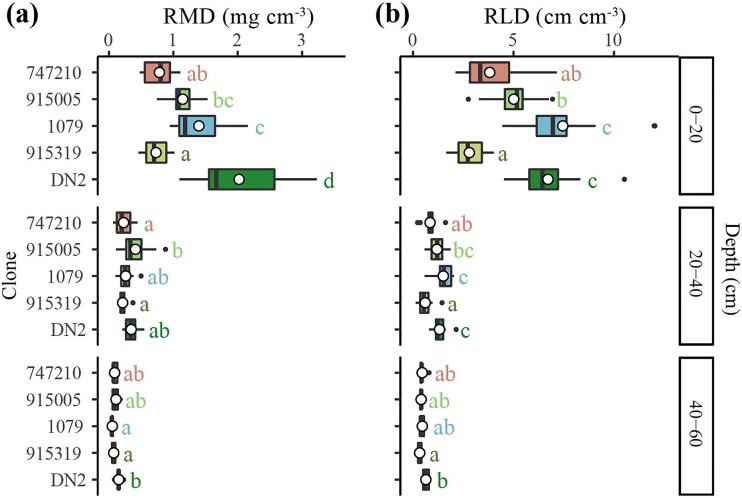
Differences in (a) root mass density (RMD) and (b) root length density (RLD) between clones at each depth. Boxplots include the median (black vertical lines) and the mean (white circles) of each root trait for each clone at each depth. Different letters indicate significant differences between clones at each depth (*P* < 0.05). Clones are arranged in ascending order of their growth rates, from top to bottom.

The most productive clone (DN2) had the greatest RMD among all the clones at the 0–20 cm depth (*P* < 0.001, Figure 4a). However, the second most productive clone (915319) had lower RMD compared to all clones except for the least productive clone (747210) at this depth (Figure 4a). RMD of the mid-productive clone (915005) was greater than those of the least productive clone (747210) and clone 915319 at the 20–40 cm depth (*P* < 0.01, Figure 4a). Furthermore, the most productive clone (DN2) had greater RMD than clones 1079 and 915319 at 40–60 cm depth (*P* < 0.01, Figure 4a). For RLD, the most productive (DN2) and the mid-productive (1079) clones distributed more root length at the 0–20 and 20–40 cm depth compared with clone 915319 and the least productive clone (747210) (*P* < 0.001 for both soil depths, Figure 4b). The most productive clones (DN2) also had greater RLD than clone 915319 at the 40–60 cm depth (*P* < 0.01, Figure 4b).

### Relationship between tree growth rates and root traits

**Figure 5:**
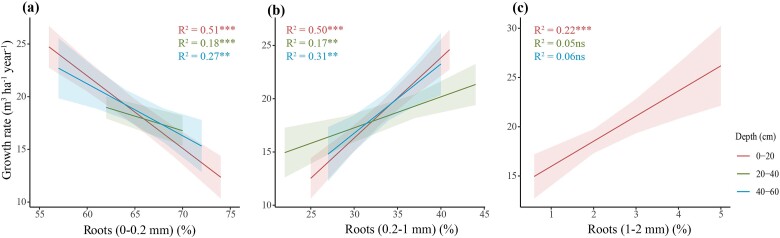
Correlation between tree growth rate and percentages in length of different root size classes. ^***^, *P* < 0.001; ^**^, *P* < 0.01; ns, not significant. Shaded areas represent the 95% confidence intervals (*n* = 60 for the 0–20 and 20–40 cm depths and *n* = 30 for the 40–60 cm depth, where n values correspond to data pairs of both tree growth rate and root traits).

**Figure 6:**
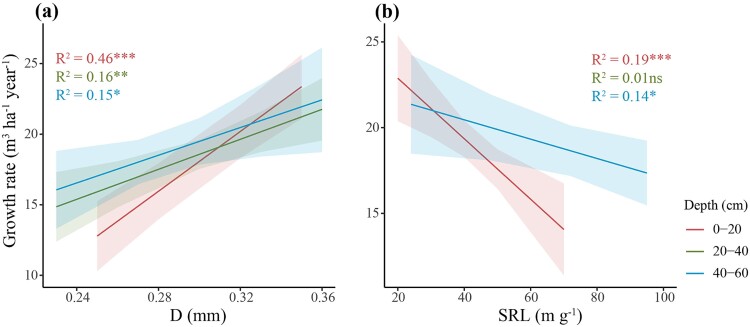
Correlation between tree growth rates and (a) average root diameter (D) or (b) specific root length (SRL). ^***^, *P* < 0.001; ^**^, *P* < 0.01; ^*^, *P* < 0.05; ns, not significant. Shaded areas represent the 95% confidence intervals (*n* = 60 for the 0–20 and 20–40 cm depths and *n* = 30 for the 40–60 cm depth, where n values correspond to data pairs of both tree growth rate and root traits).

The increase in tree growth rate was mainly linked to a reduced percentage in length of 0–0.2 mm roots relative to the total length of fine roots (VFR, *P <* 0.001), associated with an increase in root lignin concentration (*P <* 0.001) in the topsoil (0–20 cm) (Growth rate = 28.47–0.55 × (VFR) + 0.08 × (Lignin); R^2^ = 0.63). The percentage in length of 0–0.2 mm roots was also negatively correlated with tree growth rate at the 20–40 and 40–60 cm depths (Figure 5a). However, the percentage in length of 0.2–1 mm roots at the 0–20 cm depth strongly increased with aboveground growth rates (*P <* 0.001 and R^2^ = 0.50) and this correlation was also observed at the other soil depths (Figure 5b). Tree growth rates increased with the percentage of 1–2 mm roots at the 0–20 cm depth, although no correlation was found at the 20–40 and 40–60 cm depths (Figure 5c). In addition, mean diameter of fine roots was positively correlated with tree growth rate at all soil depths, with a stronger relationship for the topsoil layer (*P <* 0.001 and R^2^ = 0.46, Figure 6a). In contrast, an increase in SRL at 0–20 and 40–60 cm depths was related to lower tree growth rate (Figure 6b).

**Figure 7:**
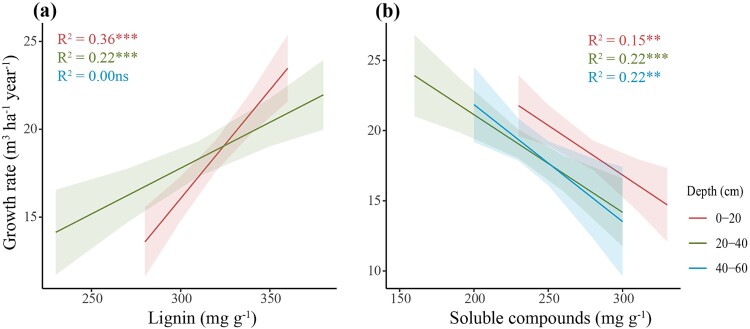
Correlation between tree growth rates and (a) root lignin or (b) root soluble compounds concentrations. ^***^, *P* < 0.001; ^**^, *P* < 0.01; ns, not significant. Shaded areas represent the 95% confidence intervals (*n* = 60 for the 0–20 and 20–40 cm depths and *n* = 30 for the 40–60 cm depth, where n values correspond to data pairs of both tree growth rate and root traits).

**Figure 8:**
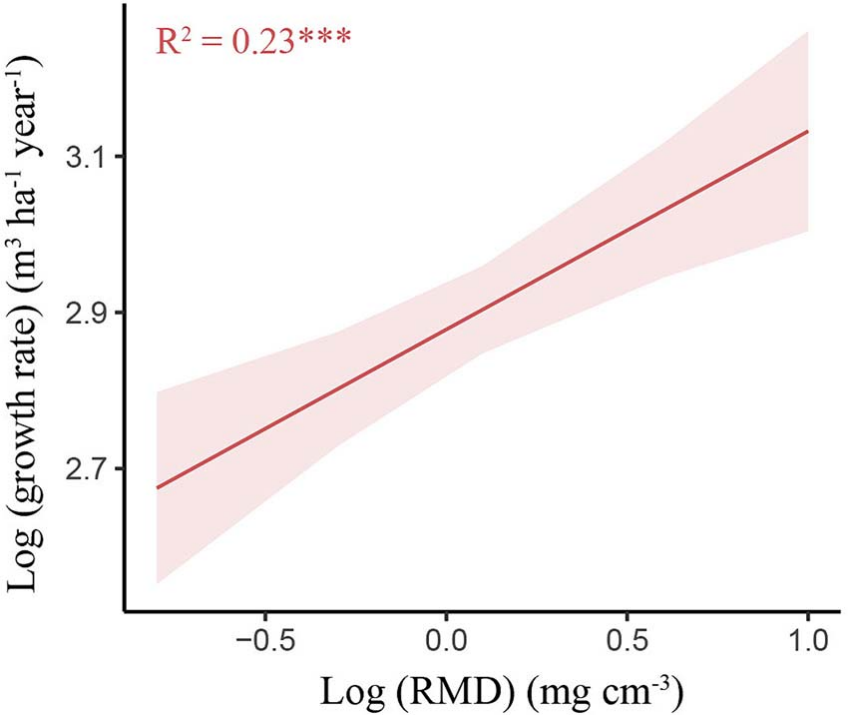
Correlation between tree growth rates and root mass density (RMD) at the 0–20 cm depth. ^***^, *P* < 0.001. Shaded areas represent the 95% confidence intervals (*n* = 60, where n values correspond to data pairs of both tree growth rate and RMD).

Root lignin concentration was also positively correlated with tree growth rate at the 20–40 cm depth (Figure 7a). Conversely, tree growth rates were negatively associated with an increase in root soluble compounds concentration at all soil depths (Figure 7b). For architectural traits, RMD was positively associated with tree growth rates at the 0–20 cm depth (*P <* 0.001, R^2^ = 0.23, Figure 8) but did not correlate with tree growth rate at the other soil depths (R^2^ = 0.00 ns and R^2^ = 0.08 ns for 20–40 cm and 40–60 cm depths, respectively). RLD exhibited a weak positive correlation with tree growth rates at the 0–20 cm depth (*P <* 0.05, R^2^ = 0.08), but had no correlations at the other depths (R^2^ = 0.04 ns and R^2^ = 0.08 ns for 20–40 cm and 40–60 cm depths, respectively).

## Discussion

### Why did the root economics spectrum (RES) not work in our fast-growing plantation?

Contrary to our expectations, SRL was not positively related to tree growth rate in our plantation and our results pointed in fact toward the opposite relationship. Consequently, our results, along with many other tree studies (e.g., Valverde-Barrantes et al. 2015; Kramer-Walter et al. 2016; Weemstra et al. 2020), did not align with the RES theory. The latter states the importance of higher SRL for nutrient foraging (Eissenstat and Yanai 1997). Several authors have argued that high SRL is more associated with soil constraints (low fertility and water stress) than with rapid tree growth (Olmo et al. 2014; Laliberté et al. 2015; Kramer-Walter et al. 2016). Since SRL did not have a positive relationship with tree growth rate, it is likely that our trees had sufficient soil nutrients and water, which could explain why the RES theory did not work in our fast-growing trees. Increasing SRL and decreasing fine root diameter also generally leads to shorter fine-root lifespan, increasing root C construction costs and probably having negative impacts on tree growth (McCormack et al. 2012; Weemstra et al. 2016). Conversely, thicker fine roots observed in the two most productive clones (DN2 and 915319) was associated with elevated tree growth, perhaps due to their longer lifespans and reduced construction costs.

Thicker, more C-rich and lignified fine roots observed in the most productive clones could also be the result of greater C assimilation during photosynthesis, enabling the construction of more transport roots and thus the exploitation of a greater soil volume. According to the surplus C hypothesis, C fixed by photosynthesis would have exceeded the C requirements of trees in these most productive clones, leading to the transfer of surplus C to roots (Prescott et al. 2020). On the contrary, a higher concentration of root soluble compounds and thinner fine roots in the less productive clones could indicate a reduced C allocation to fine roots. There would be little or no surplus C from photosynthesis in these clones, indicating that soil nutrients probably were not limiting for tree growth (Pausch and Kuzyakov 2018; Prescott et al. 2020). Otherwise, trees would have surplus fixed C if soil nutrients were limiting for tree growth, but photosynthesis continued normally (Kannenberg and Phillips 2017; Prescott et al. 2020). In this sense, the higher root N concentrations suggest that soil N was not limiting for tree growth.

Our least productive clones (747210 and 915005) not only had thinner fine roots in the upper soil layer, but also higher root N compared with the most productive clone (DN2), suggesting higher root respiration rates, as thin fine roots rich in N usually have greater metabolic activity (Eissenstat 1992; Pregitzer et al. 1998; Makita et al. 2009) and increased respiration rates (DesRochers et al. 2002; Makita et al. 2009). The increase in root respiration rates could increase C losses through root maintenance (Eissenstat 1992; McCormack et al. 2012). This could explain why clones with thinner and N-rich fine roots had lower growth rates, contrarily to our first hypothesis. Of all our fine root size categories, the fact that only the proportion in length of the very fine roots (D < 0.2 mm) showed a negative correlation with tree growth rate indicates that it could be these very fine roots that had the highest respiration rates, negatively impacting tree growth (Eissenstat 1992; Pregitzer et al. 1998; McCormack et al. 2012). We also found negative relationships between tree growth rates and soluble compounds concentrations, also pointing in the direction of increased root respiration costs, as respiration rates often increase with the concentration of root soluble compounds (Atkin et al. 2000; Lambers et al. 2002).

The positive relationships between tree growth rate and fine root diameter, as well as the proportions in length of larger fine roots (0.2–1 and 1–2 mm), could reflect greater axial transport capacity of thicker fine roots through increasing stele diameter (cylinder of vascular tissues in the root center), crucial for allocating more nutrients to aboveground components of trees (Fitter 1987; Jia et al. 2013; Kong et al. 2014). The positive effects of the 1–2 mm root class on tree growth rate nevertheless disappeared in deeper soil layers, possibly due to their lower nutrient uptake capacity and the low nutrients content of the subsoil (Jobbágy and Jackson 2000; Rumpel and Kögel-Knabner 2011). Thicker fine roots can penetrate soils more easily than thinner fine roots (Materechera et al. 1992; Hodge et al. 2009), thus facilitating the absorption of both mobile and less mobile nutrients. Furthermore, species or soil type in our experimental site could also be additional factors contributing to the inconsistency of our results with RES theory, as they can impact significantly root morphology (Materechera et al. 1992; Tobner et al. 2013). One limitation of this study is that we were unable to obtain the mass of each root size class (Root trait analysis section), and therefore unable to determine the tree growth rate–SRL relationship for each root size class.

### Increased soil exploration by fine roots can promote tree growth rate

RMD and RLD were highest in our most productive clone (DN2) and positively correlated with tree growth rate at the 0–20 cm depth, confirming our second hypothesis. The increase in these root architectural traits, in agreement with Bauhus and Messier (1999), underlines the relevance of soil exploration by fine roots to increase tree growth. Many studies in boreal and temperate forests showed that high RMD led to high aboveground growth rates, suggesting an increase in nutrients absorbed by fine roots through extended root surface area in contact with soils (Tschaplinski and Blake 1989; Hansson et al. 2013; Weemstra et al. 2020). Additionally, great RLD could facilitate the access to less mobile nutrients in soils such as phosphorus (Bauhus and Messier 1999; Ravenek et al. 2016). Similarly, in a mature forest with larger trees, trees adjusted their fine roots by increasing the size of the root system (greater RMD and RLD) rather than by increasing acquisitive fine roots (higher SRL), to match the growing demand for belowground resources (Li et al. 2019). Furthermore, the positive correlation between these traits (RMD and RLD) and tree growth rate could also indicate that fine root growth in terms of mass and length would require a significant transfer of C fixed by photosynthesis from aboveground biomass.

We noted that a positive relationship between root architectural traits and tree growth rate was only observed at the 0–20 cm depth, suggesting the importance of acquiring nutrients in the topsoil, where nutrients are usually more abundant. There was no relationship between tree growth rates and RMD and RLD in the subsoil (20–40 and 40–60 cm), likely because of the low nutrient concentration in these soil layers (Jobbágy and Jackson 2000; Rumpel and Kögel-Knabner 2011). However, the most productive clone (DN2) seemed to explore slightly more of the subsoil, with greater RMD and RLD compared with clone 915319, maybe acquiring non-negligible amounts of nutrients.

The genetic differences between clone parentage could be an important factor in the variations in RMD and RLD as the only clones which exhibited higher values of these traits (DN2 and 1079) were issued from a *Populus deltoides* Bartr. ex Marsh. cross. The clones issued from *Populus nigra* L. also had greater RMD and total root length than the clones derived from *Populus tricocarpa* Torr. & A. Gray ex Hook. (Al Afas et al. 2008), which corresponds to our observation of high RMD and RLD in clone DN2, issued from a cross between *P. deltoides* and *P. nigra*. The intensity of soil exploration, i.e., the mass or length of fine roots per unit volume of soil, appears to be species-specific and would represent an effective tree growth strategy in our fast-growing plantation.

Clone 915319 had lower RLD and RMD but higher root N concentration and root dry mass content (RDMC) compared with clone DN2 at the 0–20 cm depth, showing clear differences in their root traits even though both clones were the most productive and had similar fine root mean diameters. Therefore, they appear to have different growth strategies, where fine roots of clone 915319 contained less water but more N than those of the most productive clone (DN2) at the 0–20 cm depth. Higher N concentrations in fine roots of clone 915319 suggest that their fine root cells could contain greater amounts of proteins linked to higher root cellular activity including nutrient assimilation and transport (Ryan et al. 1996; Pregitzer et al. 1998; Jia et al. 2013). Thus, the growth strategy of the most productive clone (DN2) could be to maximize the volume of soil explored (great RMD and RLD), while that of the second most productive clone (915319) to have fewer but more absorptive fine roots to acquire nutrients efficiently. However, further analyses of root anatomy and aboveground traits (net photosynthesis, phenology, specific leaf area) are needed to better explain the differing growth strategies. This study revealed other effective tree growth strategies that are more related to soil exploration by fine roots and may help understand why RES theory has not worked in other studies of fast-growing plantations. Our findings on the links between root traits and tree growth rate could help improve species selection and management of fast-growing trees.

## Conclusion

Our study of a fast-growing plantation using various hybrid poplar clones revealed a different angle on the impact of fine root functional traits on tree growth rate. Surprisingly, the root economics spectrum theory did not align with our findings, as traits associated with resource acquisition (high SRL, thin fine roots) did not result in high tree growth rates. However, mean diameter of fine roots was positively related to tree growth rate, probably due to the extended lifespan and reduced respiration rates of these fine roots, decreasing root construction and maintenance costs, respectively. The thicker, more lignified fine roots observed in the most productive clones could also be the result of greater C assimilation during photosynthesis, with surplus C being transferred to roots. The strategy of enhancing soil exploration intensity linked with greater RMD also proved effective in promoting tree growth within our plantation, where rapid growth was accompanied by substantial fine root growth in terms of diameter and mass to increase the root system size and nutrient acquisition. Extending this study to encompass lifespan and respiration rates of fine roots could provide further insights into relationships between tree growth rates and root traits.

## Supplementary Material

Fig_S1_(a)_tpae120

Fig_S1_(b)_tpae120

Supplementary_data_tpae120

## Data Availability

The data underlying this article are available in the Zenodo repository [https://doi.org/10.5281/zenodo.10919650].
