# Supplementary figures and images for: How is tree growth rate linked to root functional traits in phylogenetically related poplar hybrids?
Source: Tree Physiol. 2024 Sep 16;44(10):tpae120. doi: 10.1093/treephys/tpae120 (PMC11469761; doi:10.1093/treephys/tpae120)

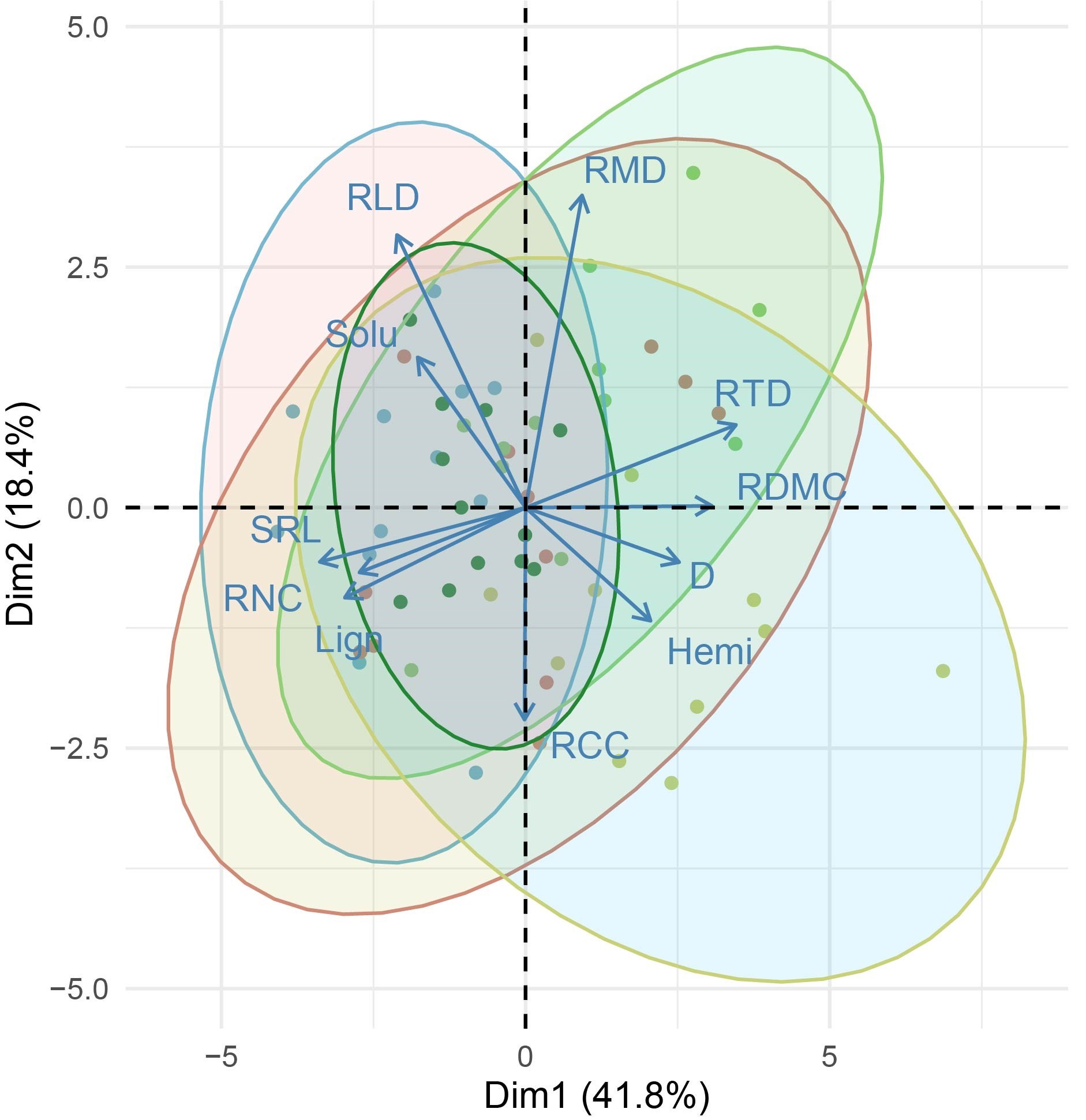

Supplement: Fig_S1_(a)_tpae120 [file fig_s1_(a)_tpae120.jpeg]

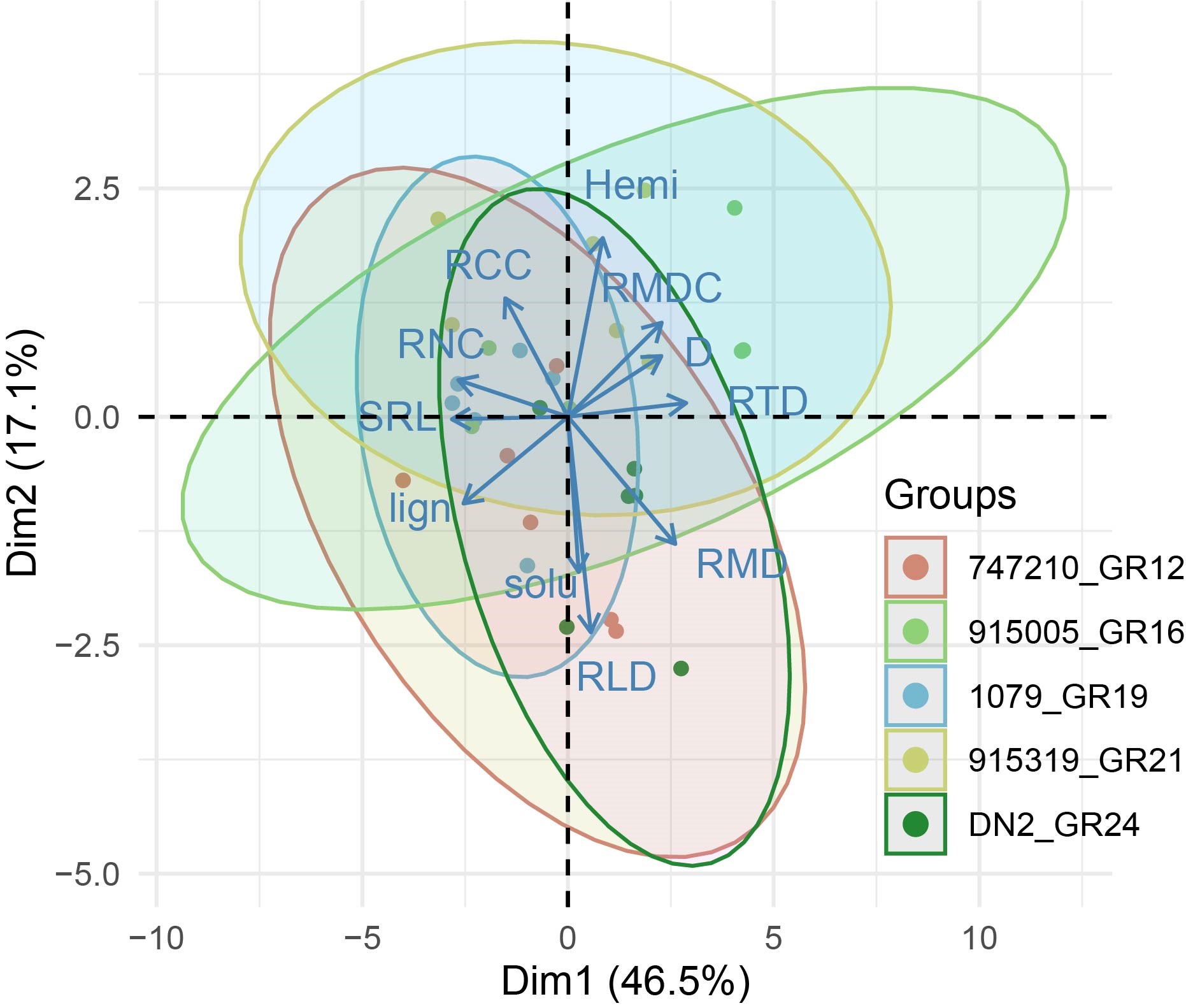

Supplement: Fig_S1_(b)_tpae120 [file fig_s1_(b)_tpae120.jpeg]
