# Supplementary material for: How is tree growth rate linked to root functional traits in phylogenetically related poplar hybrids?
Source: Tree Physiol. 2024 Sep 16;44(10):tpae120. doi: 10.1093/treephys/tpae120 (PMC11469761; doi:10.1093/treephys/tpae120)
Supplement: Supplementary_data_tpae120 [file supplementary_data_tpae120.docx]

**Supplementary data**

Article title: How is tree growth rate linked to root functional traits in phylogenetically related poplar hybrids?

Authors: Toky Jeriniaina Rabearison, Vincent Poirier, Jérôme Laganière, Annie DesRochers

Additional data in this article include:

**Fig. S1** PCA of fine-root traits at 20-40 (A) and 40-60 cm (B) depths

**Table S1** Model selection on relationships between root traits and tree growth rates at the 0-20 cm depth according to AIC

**Table S2** Model selection on relationships between root traits and tree growth rates at the 20-40 cm depth according to AIC

**Table S3** Model selection on relationships between root traits and tree growth rates at the 40-60 cm depth according to AIC


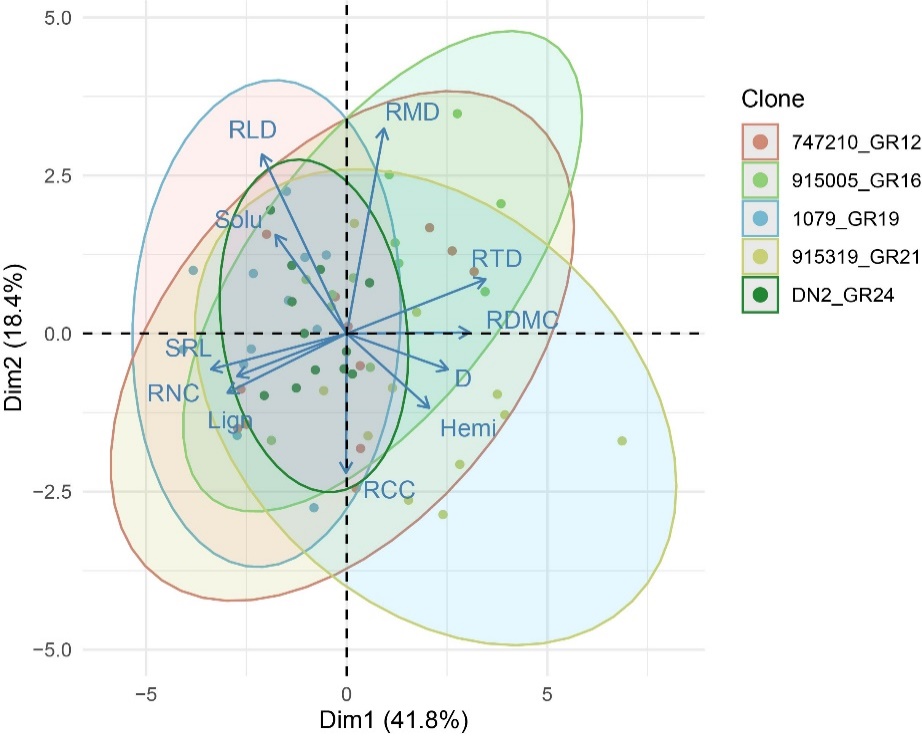

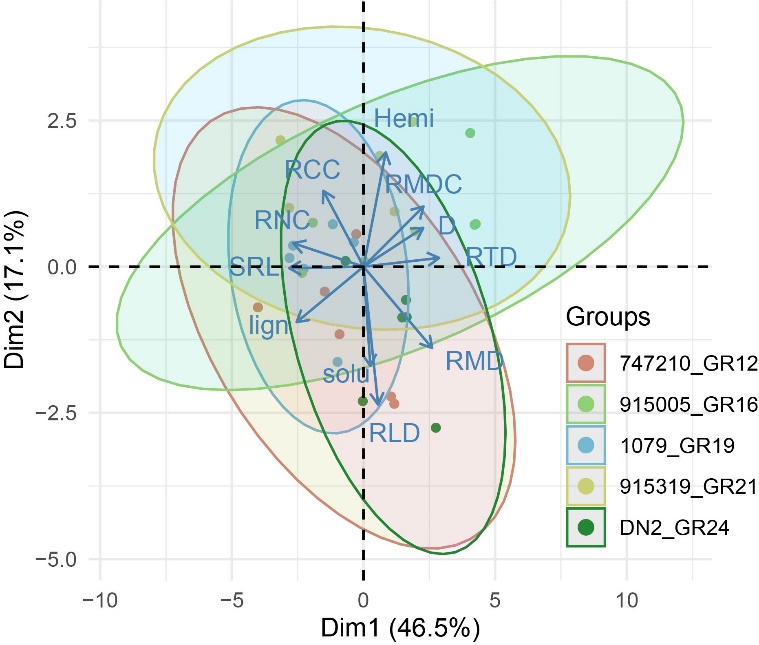


(A)

(B)

**Fig. S1** PCA of fine-root traits at 20-40 (A) and 40-60 cm (B) depths. Each color represents one clone that is labeled with its parentage coding and growth rate (GR). D: average root diameter, Hemi: root [hemicellulose], Lign: root [lignin], RCC: root [carbon], RDMC: root dry matter content, RLD: root length density, RMD: root mass density, RNC: root [nitrogen], RTD: root tissue density, Solu: root [soluble compounds] and SRL: specific root length.

**Table S1** Model selection on relationships between root traits and tree growth rates at the 0-20 cm depth according to AIC. AICc: Akaike Information Criterion, D: average root diameter, RCC: root [carbon], RDMC: root dry matter content, RLD° root length density, RMD: root mass density, RNC: root [nitrogen], RTD: root tissue density and SRL: specific root length

| Root trait | Estimate | *P* | R^2^ | AICc | ΔAICc |
| --- | --- | --- | --- | --- | --- |
| D | 105.77 | **<0.001** | 0.46 | 320.77 | 0 |
| Lignin | 0.12 | **<0.001** | 0.36 | 326.08 | 5.31 |
| RMD | 3.74 | **<0.001** | 0.25 | 335.08 | 14.31 |
| RCC | 0.27 | **<0.001** | 0.21 | 338.16 | 17.39 |
| SRL | -0.18 | **<0.001** | 0.19 | 340.07 | 19.3 |
| Soluble compounds | -0.07 | **<0.01** | 0.15 | 342.48 | 21.71 |
| RNC | -0.99 | **0.02** | 0.09 | 346.68 | 25.91 |
| RLD | 0.53 | **0.03** | 0.08 | 347.48 | 26.71 |
| Hemicellulose | -0.06 | 0.32 | 0.02 | 351.37 | 30.6 |
| RDMC | -0.01 | 0.5 | 0.00 | 351.9 | 31.13 |
| Cellulose | -0.01 | 0.56 | 0.00 | 352.01 | 31.24 |
| RTD | 4.39 | 0.71 | 0.00 | 352.14 | 31.44 |

**Table S2** Model selection on relationships between root traits and tree growth rates at the 20-40 cm depth according to AIC. AICc: Akaike Information Criterion, D: average root diameter, RCC: root [carbon], RDMC: root dry matter content, RLD° root length density, RMD: root mass density, RNC: root [nitrogen], RTD: root tissue density and SRL: specific root length

| Root trait | Estimate | *P* | R^2^ | AICc | ΔAICc |
| --- | --- | --- | --- | --- | --- |
| Lignin | 0.05 | **<0.001** | 0.22 | 327.46 | 0 |
| Soluble compounds | -0.07 | **<0.001** | 0.22 | 327.77 | 0.32 |
| D | 53.08 | **<0.01** | 0.16 | 331.68 | 4.22 |
| RMDC | -0.04 | **<0.01** | 0.11 | 334.86 | 7.41 |
| Hemicellulose | -0.10 | **0.02** | 0.10 | 335.8 | 8.34 |
| RNC | -0.74 | **0.04** | 0.07 | 337.23 | 9.78 |
| RCC | 0.10 | **0.04** | 0.06 | 337.57 | 10.11 |
| RTD | -7.72 | 0.07 | 0.06 | 338.23 | 10.78 |
| RLD | 1.74 | 0.13 | 0.04 | 339.35 | 11.9 |
| RMD | 2.30 | 0.53 | 0.00 | 341.23 | 13.77 |
| SRL | -0.02 | 0.55 | 0.01 | 341.26 | 13.81 |
| Cellulose | 0.00 | 0.9 | 0.00 | 341.61 | 14.16 |

**Table S3** Model selection on relationships between root traits and tree growth rates at the 40-60 cm depth according to AIC. AICc: Akaike Information Criterion, D: average root diameter, RCC: root [carbon], RDMC: root dry matter content, RLD° root length density, RMD: root mass density, RNC: root [nitrogen], RTD: root tissue density and SRL: specific root length

| Root trait | Estimate | *P* | R^2^ | AICc | ΔAICc |
| --- | --- | --- | --- | --- | --- |
| Soluble compounds | -0.08 | **<0.01** | 0.22 | 174.06 | 0 |
| D | 49.02 | **0.03** | 0.15 | 176.62 | 2.55 |
| SRL | -0.06 | **0.03** | 0.15 | 176.75 | 2.68 |
| RLD | 6.83 | 0.13 | 0.08 | 179.13 | 5.06 |
| RMD | 21.07 | 0.14 | 0.08 | 179.14 | 5.08 |
| Cellulose | 0.03 | 0.23 | 0.05 | 179.92 | 5.86 |
| RNC | -0.36 | 0.39 | 0.03 | 180.65 | 6.59 |
| Hemicellulose | 0.05 | 0.49 | 0.02 | 180.94 | 6.87 |
| RTD | 5.37 | 0.52 | 0.01 | 181 | 6.93 |
| Lignin | 0.00 | 0.68 | 0.00 | 181.24 | 7.17 |
| RDMC | 0.00 | 0.92 | 0.00 | 181.4 | 7.33 |
| RCC | 0.00 | 0.96 | 0.00 | 181.41 | 7.34 |
